# How and why eLife selects papers for peer review

**DOI:** 10.7554/eLife.100571

**Published:** 2024-07-23

**Authors:** eLife Editorial Leadership, Timothy E Behrens, Yamini Dalal, Diane M Harper, Detlef Weigel, eLife Senior Editors, Olujimi A Ajijola, Amy H Andreotti, Sofia J Araújo, Utpal Banerjee, Balram Bhargava, Yanchao Bi, Christian Büchel, Felix Campelo, Albert Cardona, Kathryn Cheah, Lu Chen, Murim Choi, Laura Colgin, Jonathan A Cooper, Qiang Cui, Floris de Lange, Claude Desplan, Volker Dötsch, Wafik S El-Deiry, Eduardo Franco, Michael J Frank, Wendy S Garrett, Joshua I Gold, Silke Hauf, Christopher L-H Huang, John Huguenard, David E James, Bavesh Kana, Pankaj Kapahi, Andrew J King, Jürgen Kleine-Vehn, Benoît Kornmann, Caigang Liu, Huan Luo, Merritt Maduke, Tamar Makin, André F Marquand, Adèle L Marston, Pramod Mistry, Alan Moses, Sacha B Nelson, Tony Ng, Päivi Ojala, George H Perry, Panayiota Poirazi, Lynne-Marie Postovit, Sohini Ramachandran, Sergio Rasmann, Satyajit Rath, Jonathan Roiser, David Ron, Carla V Rothlin, John W Schoggins, Meredith C Schuman, Barbara G Shinn-Cunningham, Dolores Shoback, Lois Smith, Dominique Soldati-Favre, Didier Stainier, Lori Sussel, Kenton J Swartz, Michael Taffe, Tadatsugu Taniguchi, K VijayRaghavan, Aleksandra Walczak, Kate Wassum, Richard M White, Ma-Li Wong, Wei Yan, Tony Yuen, eLife Early Career Advisory Group, Mayank Chugh, Laura Han, Sarah Marei, Regina Mencia, Divyansh Mittal, Elizabeth Ochola, Facundo Romani, Lynn Yap

**Keywords:** scientific publishing, peer review, preprints, research assessment, research communication

## Abstract

When deciding which submissions should be peer reviewed, eLife editors consider whether they will be able to find high-quality reviewers, and whether the reviews will be valuable to the scientific community.

eLife is working to promote a culture in which the actual content of a paper is more important than the name of the journal in which it is published. A culture in which scientific research is first disseminated as a preprint and then assessed and evaluated in depth by experts. This is why papers published in eLife include the preprint itself, an eLife assessment and Public Reviews written by the editor and reviewers, and a response from the authors (if available).

By making the views of expert editors and reviewers an integral part of the published paper, we hope to improve the way that scientific research is assessed and evaluated. Where readers are experts, they can assess the work for themselves. If not, they can rely on our Public Reviews, which go into the strengths and weaknesses of the paper in detail. And if they require a concise critique, they can read the eLife assessment, which summarises the significance of the findings reported in the paper (on a scale ranging from useful to landmark) and the strength of the evidence (inadequate to exceptional).

This approach of moving beyond binary accept/reject decisions and more fully conveying the views of expert reviewers has many advantages. It helps those who have to evaluate researchers and their work to make better-informed decisions about funding, hiring and promotion. It means that reviewers cannot prevent the publication of a paper that has been selected for peer review, which allows authors to engage more freely with reviewers during the revision process, without worrying about having to start over at a new journal. It also ensures that comments from the reviewers are valued as an integral part of the scientific literature. Overall, this approach enables the rapid and scholarly dissemination of new scientific knowledge in a way that permits the views and constructive criticisms of expert reviewers to be openly considered by both authors and readers. More information is available on the eLife website: elifesciences.org/about/peer-review.

If this is what we want, why do we only review some papers and not others? Achieving our goals requires us to make tough choices about which submissions to prioritise for in-depth review. Our mission will succeed only if we ensure that our unique offering – Public Reviews plus an eLife assessment – remains high quality, and that our reviews are read and have influence.

## Finding the right reviewers

Recruiting good reviewers is difficult, and it is getting more difficult as the volume of published work increases year-on-year. Importantly, it is easier to find reviewers, particularly those with the deep expertise we need, for work that is interesting and rigorous. People simply prefer to review work that is of interest to them, and the same is true when it comes to finding editors to oversee the review process.

So there is a clear tension: we would like the outputs of the eLife peer-review process to be of the highest quality, but preparing such assessments is easier for work that editors and reviewers are willing to engage with. Therefore, one clear reason for why we only peer review some submissions is to ensure the quality and rigour of what we produce.

## Focusing our efforts

We must continue to focus our efforts on those papers where the outputs of the eLife peer-review process are most valuable. This is not the same as deciding which papers are “the best”. For example, there might be value in reviewing work that is controversial in a particular field of research. However, it does mean concentrating on papers where we believe the scientific content will be most inspiring to eLife readers.

Just as editors and reviewers prefer to review work that is of interest to them, as readers we scientists pay more attention to reviews of work that has the potential to change the way we think about a question. Therefore, if we are going to change scientific publishing for the better, we will do so faster by focusing our efforts on such papers.

## How it works

The peer-review process at eLife is overseen by a team of over 70 Senior Editors and more than 700 Reviewing Editors. Every paper submitted to the journal is assigned to a Senior Editor, who generally asks a small number of Reviewing Editors about the submission. The Reviewing Editors offer their scientific views on the paper, and there is an open discussion about whether to review the paper, using the reasoning laid out above. Editors are considering whether the work is of substantial interest, whether they will be able to find high-quality reviewers, and whether the reviews will be valuable to the scientific community.

If they decide to proceed, and if one of the Reviewing Editors agrees to handle the submission, they initiate the labour of love that is peer review. Once the reviews have been received, the Reviewing Editor and the reviewers craft the eLife assessment, which is vetted by the Senior Editor before publication. This assessment, together with the Public Reviews, helps readers to better understand the new findings, their validity, and their relevance to the broader scientific community. If this approach were adopted more widely, it would mean that a reader could judge the work in a given paper on its own merits, irrespective of where it was published.

